# Detection of HHV-5 HHV-6a HHV-6b and HHV-7 in the urine: potential use as a non-invasive diagnostic tool for immune profiling

**DOI:** 10.1186/s12979-024-00490-9

**Published:** 2024-11-29

**Authors:** Shelia Govind, Pierre Olivier Lang, Alexander Bürkle, María Moreno-Villanueva, Claudio Franceschi, Miriam Capri, Jurgen Bernard, Birgit Weinberger, Beatrix Grubeck-Loebenstein, Simone Fiegl, Efstathios S. Gonos, Ewa Sikora, Eugène Jansen, Martijn E. T. Dollé, Tilman Grune, Nicolle Breusing, Richard Aspinall

**Affiliations:** 1 Medicines and Healthcare Products Regulatory Agency (MHRA), South Mimms Laboratories, Potters Bar, UK; 2 Genolier Clinic, Genolier, Switzerland; 3 Molecular Toxicology Group, Department of Biology, University of Konstanz, Box 628, Konstanz, 78457 Germany; 4 Human Performance Research Centre, Department of Sport Science, University of Konstanz, Box 30, Konstanz, 78457 Germany; 5 Institute of Biogerontology, Lobachevsky State University, Nizhny Novgorod, Russia; 6 Department of Medical and Surgical Sciences, University of Bologna-Alma Mater Studiorum, Bologna, Italy; 7 Alma Mater Research Institute On Global Challenges and Climate Change (Alma Climate), University of Bologna, Bologna, Italy; 8 BioTeSyS GmbH, Essliongen, Germany; 9 Institute for Biomedical Aging Research, Universität Innsbruck, Innsbruck, Austria; 10 UMIT TIROL, Innsbruck, Austria; 11 NHRF, Institute of Biology, Athens, Greece; 12 Polish Academy of Sciences, Warsaw, Poland; 13 Centre for Health Protection, National Institute for Public Health and the Environment, PO Box 1, 3720 BA Bilthoven, Utrecht, The Netherlands; 14 National Institute for Public Health and the Environment, Utrecht, The Netherlands; 15 Department of Molecular Toxicology, German Institute of Human Nutrition Potsdam-Rehbruecke (DIfE), Nuthetal, 14558 Germany; 16 Department of Applied Nutritional Science/Dietetics, Institute of Nutritional Medicine, University of Hohenheim, 70599 Stuttgart, Germany; 17 Centre for Intelligent Healthcare, Coventry University, Priory Street, COVENTRY, CV1 5FB, Conventry, UK

**Keywords:** Human Herpes Virus, T cell receptor excision circles

## Abstract

Decline in immune function with age has been studied extensively, but approaches to immune restoration have been hampered by the lack of simple methods of identifying individuals whose immune system is in decline. Our approach has been to identify individuals whose immune decline has led to a loss of control of common latent viral infections and their consequent reactivation. Viruses excreted in urine were detected and quantified and we believe this approach could provide a 'surrogate marker' for identifying immune compromised individuals.

Here we report the detection of human herpes virus (HHV) 5, 6a, 6b and 7 in the urine of healthy individuals over a wide age range and their correlation with T cell receptor excision circle (TREC) data. The results did not show a clear correlation between TREC values and the detection of individual specific viruses or viral load values when measured singly.

However, a correlation was found between low TREC values and the detection of several different human herpes viruses in the urine in males. We present evidence suggesting that for males, the detection of three or more different human herpes viruses in the urine could identify individuals with declining immune function as evidenced by their significantly lower TREC levels.

## Introduction

Of the more than 100 known herpesviruses, 9 are known to infect humans (human Herpes viruses – HHV) and these have been divided into three families: – α-HHV (herpes simplex virus types 1 and 2) or HSV-1 and HSV-2 and varicella-zoster virus (VZV or HHV-3;); β-HHV which includes cytomegalovirus (CMV or HHV-5); human herpesvirus 6 variants A and B (HHV-6A and B); and HHV-7; and γ-HHV which includes Epstein-Barr virus (EBV or HHV-4) and Kaposi's sarcoma virus (HHV-8). HHVs have a four-layered structure consisting of a core containing the large, double-stranded DNA genome of up to approximately 250kD which is enclosed by an icosapentahedral capsid composed of capsomers contained within a protein coat covered by a lipid bilayer containing glycoproteins [1].

HHVs are known for their ability to establish lifelong infections within specific tissues, which are characteristic for each virus and in selected cell types. The latent virus may reactivate at any time, but the precise mechanism of reactivation is unknown. β-HHV are commonly associated with infection in younger individuals. Most children are infected with HHV-6 and HHV-7 by 3 years of age, but for HHV-5 the incidence is somewhat different with the timing of acquisition varying considerably [2–4]. Despite this initial difference, by the time an individual reaches 75–80 the likelihood of being infected is high with 80–90% of the population at this age being seropositive [5, 6].

Whilst the period after initial infection is often free of symptoms, control of the β-HHV depends on an ongoing immune response to reduce the frequency and consequences of their reactivation. When immune responses are compromised for example in transplant patients, virally associated disease may ensue [7] and virus is detectable in the urine [8]. Several studies have linked infection with HHV-5 with a significantly increased risk for all-cause mortality [9] with an increased incidence of cardiovascular disease [10] and with considerable change to the immune system including an increasing number of CMV-specific T cells and a suppression of naïve cell numbers [11]. The effect of HHV-5 on the immune system in older individuals has led to its linkage with poorer immune responses [12].

Age-associated dysregulation of the immune system is often termed immunosenescence, which is a broad term encompassing a series of age-related changes in both the adaptive and innate arms of the immune system and leaves an individual with a sub-optimal immune response. Factors which have been suggested to contribute to this age-associated immune decline include changes in function and number of Langerhans cells [13]; dendritic cells [14]; alterations in T cell [15], B cell [16] and neutrophil [17] function; reduction in the T and B cell repertoires[18, 19] and in T cell [20] and B cell [21] production. These changes, in addition to poor nutritional status [22] and comorbid conditions [23] all contribute towards an individuals’ inability to make an adequate protective response following vaccination or exposure to a potential pathogen or control of latent viral burden.

Not all older individuals show immune decline according to a specific tempo. For example, influenza vaccination studies reveal that many older individuals when vaccinated with the trivalent vaccine make responses whose specific anti-influenza antibody titres are lower than seen with younger individuals and may thus provide less protection from infection [24]. In a study on responses to vaccination with hepatitis B on a cohort of 45 individuals whose average age was 74, 26 failed to produce antibody responses which would provide a protective response [25]. These studies illustrate the issue that in older people it is not possible to predict who will respond to vaccination by making a protective antibody response. There are currently no clear simple assays which indicate whether an older individual will produce a response to vaccination which would indicate full protection.

Previous work suggested that CMV (HHV-5) could be detected in the urine of older individuals and the authors speculated that this may indicate a change in the management of the virus such that the infection becomes chronic rather than latent; linking it to a loss of viral control [26]. This would be in line with other work on the herpes viruses which shows that as the immune system declines with age there is reactivation and subsequent disease as with VZV (HHV-3) and the appearance of shingles [27] or HHV-4 and the appearance of lymphoma [28]. With this in mind and the need to provide a means of immune profiling individuals [29], we carried out a pilot study on samples gathered from healthy individuals across Europe at different ages to try to determine whether we could identify viral DNA in the urine of these individuals and whether the presence of the virus could be related to their immune status.

## Materials and methods

### Study population

The study population comprised 700 randomly recruited age-stratified individuals from the general population covering the age range 35–74 years (“RASIG”) which were part of a larger cross-sectional study cohort recruited by the MARK-AGE consortium [30–33] (http://www.mark-age.eu). Equal numbers of males and females were recruited into 1 of 8 age classes 35–39, 40–44, 45–49, 50–54, 55–59, 60–64, 65–69, and 70–74 years from several different geographical regions of Europe.

### Ethics, sample collection and storage

The study population was drawn from different regions of Europe with recruitment centres in Germany, Poland, Greece, Austria, Italy and Finland. The Local Research Ethics Committees of the respective recruiting centres provided ethical approval for the Mark-Age project which was registered with the German Clinical Trials Register (DRKS00007713). Following informed consent, biological samples including blood and urine were collected from participants observing at least 8 h fasting (except water and medication). Anthropometric, clinical and social data were also collected. Urine (containing ~ 1 mM sodium azide) and PBMC samples were stored immediately at −80°C. Following overnight storage PBMCs were transferred to liquid nitrogen (−196°C) until shipment to the BIOBANK (Hohenheim, Germany) on dry-ice. Biological samples were received by the Biobank under a primary subject code (PSC) and re-aliquoted and relabelled with a secondary subject code (SSC) and distributed for analyses. Upon receipt, all urine and PBMC samples were maintained at −80°C and −150°C, respectively, until further analysis. Participant details (age, sex, etc.) remained unknown throughout the analysis.

### Urine sample preparation

Urine samples (3 mL) were thawed at 4⁰C before concentration using Ultrafiltration Vivaspin 6 spin columns (Generon, UK) supplemented with 3 ml of sterile PBS. Columns were centrifuged 20–30 min at 4000 g at 4⁰C until the volume had reduced to ~ 200 µl. Centrifugation was repeated following the addition of 6 ml of sterile PBS until the sample was concentrated down to 140 µl. Viral nucleic acid extraction was performed on the automated extraction unit QIAcube using the Viral RNA QIAamp extraction kit (QIAGEN) according to the manufacturer’s specifications. Sample elution volume was 60 µl and extracted DNA samples were stored at −80⁰C.

### PBMC sample preparation for sjTREC (signal joint TREC) determination

Cryovials were briefly thawed in a 37⁰C water bath to which 500 µl of cell culture medium (90% RPMI, 10% FCS) was added. PBMC suspension was transferred to a 15 ml falcon tube to which 1 ml, then 2 ml and then 4.0 ml of thawing medium was added in a drop wise fashion. Cells were centrifuged at 200 g for 10 min at low temp (5⁰C). Cell pellets were gently resuspended in 1.2 ml of medium and placed on ice. Cell number and viability was determined using the Countess Cell counter (Invitrogen). A minimum of ~ 1 × 10e5 cells were used to determine the CD3^+^% of the PBMC sample using PE- conjugated anti CD3 antibody (BD Biosciences) by Flow cytometry (Accuri C6 sampler, BD Biosciences). Cell pellets were resuspended in 100 µl staining buffer containing a 1:25 dilution of PE- conjugated anti CD3 antibody and stored at 4⁰C for 30–60 min. Cell were washed with staining buffer before fixing in 1% Paraformaldehyde (PFA), Samples were stored for up to a week before analysis on C6 sampler. 1 ml aliquots of PBMCs were centrifuged and resuspended into 200 µl of sterile PBS and DNA extractions were performed on the QIAcube using the QIAamp DNA mini according to manufacturer’s recommendation. Samples were eluted in 100 µl of AE and DNA concentration was determined using the Picodrop. Samples were stored at −80⁰C.

### Plasmid construct preparation for absolute qPCR determination of β-HHV

HHV-6A strain GS and HHV-6B strain Z9 viral DNA (Tebu Bio) were used as templates for insert amplification of region U83 (138 bp) of HHV-6A, using forward; 5’-ATGGCTATCGGATTTATATGTAGT-3’ and reverse 5’-GCTCTGTTTTCCCAGGTA-3’ primers, and region U67 (221 bp) of HHV6B using forward 5’-TGATCGAAACGCCTACACAG-3’ and reverse 5’- AATGTACGTCCCCGAAATGG-3’ primers adapted from (Ward, Leong et al. 2007). Region U84 (127 bp) of HHV-7 was amplified using DNA extracted from HHV-7 strain J1 infected SUPT-1 cells (kindly provided by HHV-6 foundation) with forward 5’-TTCATGTAGATCGCGGGCTTT-3’ and reverse 5’- ATTTCCGGAATGTAGCCAACAA primers (Ferreira et al., 2011). Inserts were amplified using standard conditions with GoTaq polymerase (Promega) and purified before ligation into pGEM-T easy vector (Promega). Competent bacterial strain JM109 cells were transformed with the ligation mix and positive transformants were identified using blue/white selection. Positive clones were confirmed with PCR amplification using purified plasmid DNA and further verified by sequencing (GATC). The CMV (HHV-5) construct is as described in (Atkinson et al., 2009) containing a 147 bp fragment amplified by forward (gB2) 5’-TCGACG GTG GAG ATA CTG CTG AGG-3’ and reverse (gB1) 5’-GAG GAC AAC GAA ATC CTG TTG GGC A-3’ primers cloned into the TOPO expression vector (Invitrogen).

### qPCR quantification

Absolute quantification of viral copy number in urine was performed using a log linear 10^8^–10^1^ standard curve using a plasmid construct of each virus of interest. qPCR reactions were prepared in triplicate using the automated liquid handling platform QIAgility and performed on the Rotorgene Q (Qiagen). Standard cycling conditions were hot start at 95 ⁰C for 15 min, and 40 cycles of 95 ⁰C for 15 s, 55–60 ⁰C for 30 s. Annealing temperatures for each viral construct were as follows HHV-6A 55.7⁰C, HHV-6B 58⁰C, HHV-7 58⁰C, CMV 57.7⁰C), followed by extension at 72 ⁰C for 30 s. A 10 µl total reaction mix comprised of 5 µl of 2 × Quantitect SYBR (Qiagen), 0.5 µM forward and reverse primers, 2 µl of extracted urine sample and 2 µl of water. The specificity of the qPCR products were validated by melt-curve analysis using the Rotorgene Q software to confirm single product amplification. Prior to sjTREC determination, PBMC samples were normalised with albumin housekeeping gene to ensure DNA quantity and integrity as previous described [44]. qPCR reactions were performed as described above using total genomic DNA equivalent to 50 ng/reaction.

### Statistics

The results of the descriptive analysis are presented for numerical variables in the form of averages, sample sizes and percentages were calculated for categorical outcomes. Statistical tests used for the comparative analysis were chosen according to the type of variable, the sample size under consideration, and the number of groups compared.

## Results

### Detection of viral DNA in urine

**Fig. 1:**
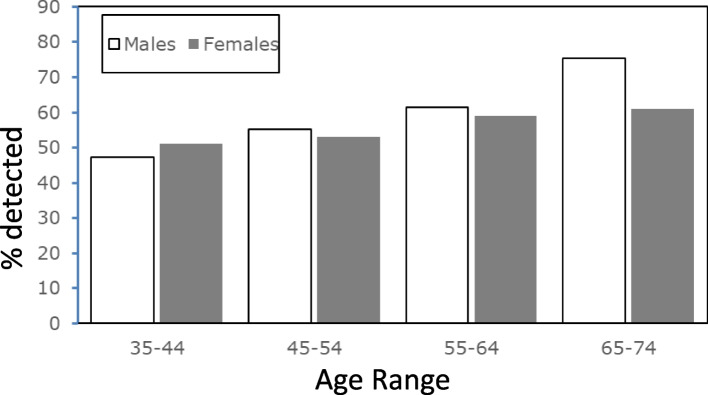
Percentage of positive samples in the urine of males (unshaded bars) and females (shaded bars) between the ages of 35 and 74

We were able to detect β-hhv viral dna in the urine of both men and women between the ages of 35 and 74 using the real time pcr assay system. Of the 282 female samples tested, we could detect virus in 160 (57%) of the samples and the age spread of the positive samples is shown in Fig. 1. For the male samples, virus could be detected in 165 (59%) of the 278 samples tested. Both males and females showed an overall age-related increase in the number of samples in which virus could be detected. Across the different types of HHV detected we could find no dramatic rise in any of the specific types with age (Tables 1 and 2).

**Table 1:** Females

Age range	n	undetectable	%	positive	%	HHV5	%	HHV6a	%	HHV6b	%	HHV7	%
35–44	75	34	45	41	55	24	59	16	39	3	7	19	46
45–54	68	32	47	36	53	22	61	12	33	10	28	15	42
55–64	68	28	41	40	59	30	75	21	53	6	15	16	40
65–74	71	28	39	43	61	22	51	15	35	6	14	21	49
total	282	122	43	160	57	98	61	15	9	25	16	71	44

**Table 2:** Males

Age range		n		undetectable			%	positive		%		HHV5		%		HHV6a		%		HHV6b		%		HHV7		%	
35-44		70		37			53	33		47		19		58		13		39		4		12		10		30	
45-54		69		31			45	38		55		24		63		16		42		5		13		12		32	
55-64		78		30			38	48		62		24		50		18		38		4		8		21		44	
65-74		61		15			25	46		75		25		54		14		30		9		20		17		37	
total		278		113			41	165		59		91		56		61		37		22		13		60		36	

**Fig. 2:**
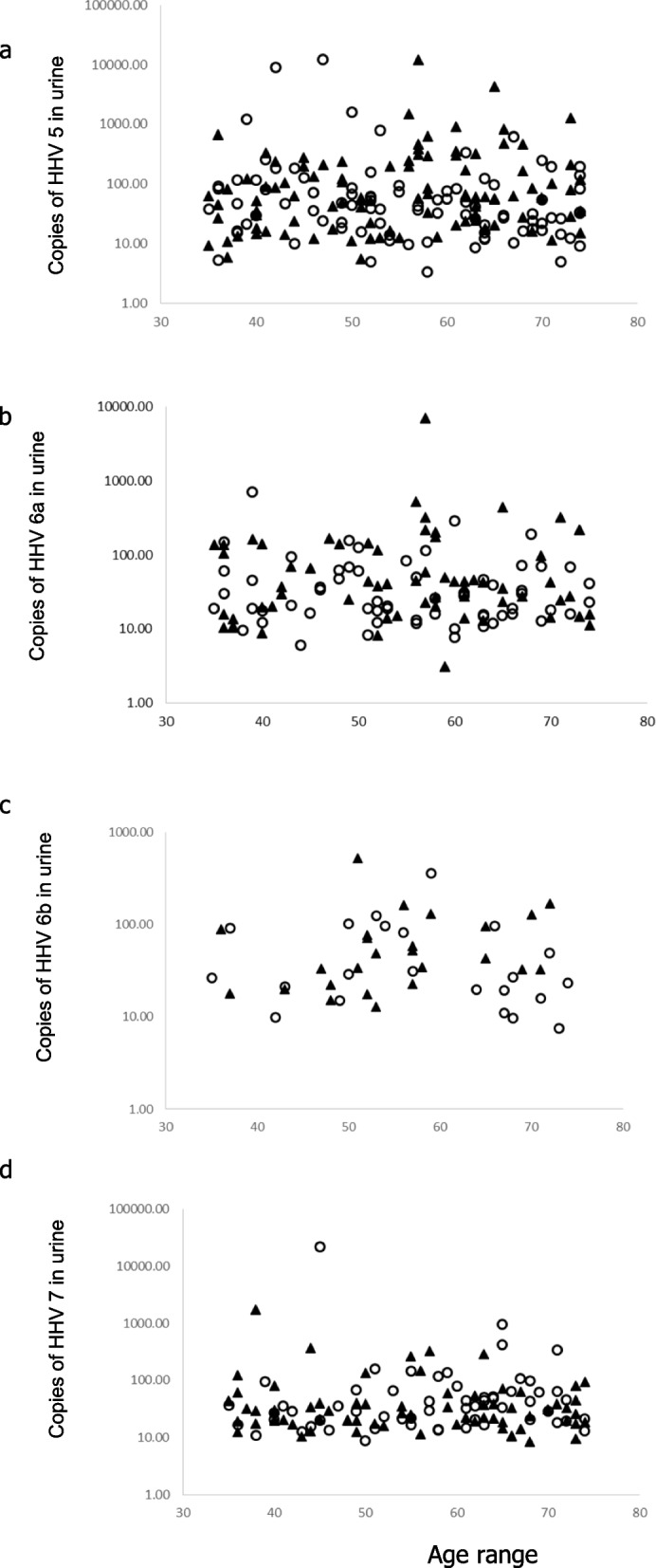
Human herpes virus in the urine of males (◯) or females (▲). Graphs show (**a**) HHV-5, **b** HHV-6a (**c**) HHV-6b and (**d**) HHV-7

With more older individuals showing detectable virus DNA in their urine we hypothesised that with increasing age there would be an increasing likelihood of immune decline and so the potential for increased viral load in the urine. We tested for a relationship between age and viral load in the urine and the results shown in Fig. 2 reveal no clear association between the amount of viral DNA detected in the urine and the age of the person from whom the sample was obtained.

### sjTREC

**Fig. 3:**
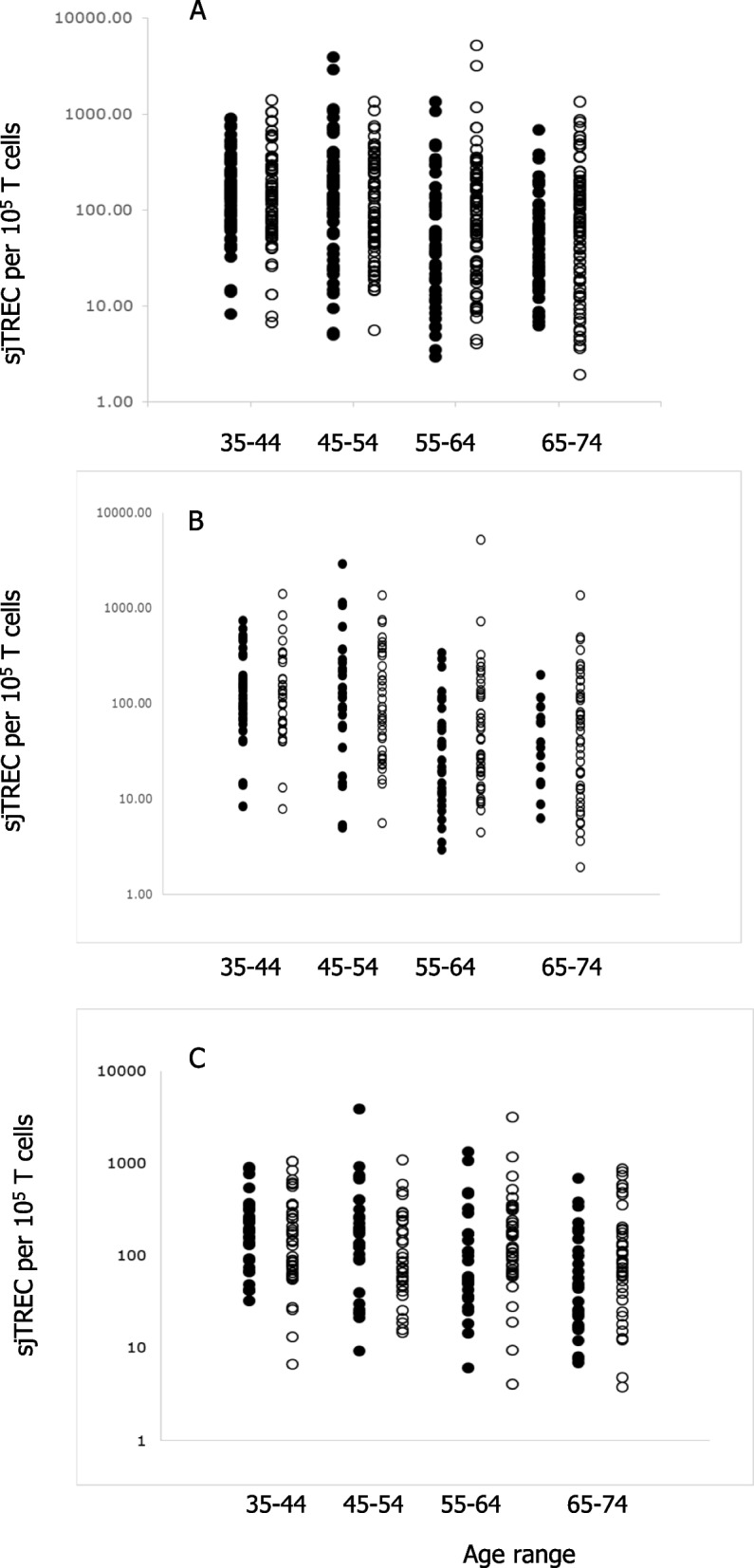
The number of sjTREC /100000 T cells for (**a**) all samples or (**b**) males or (**c**) females comparing positives (◯) with samples where virus was not detectable (●) according to age

Analysis of the values obtained for sjTREC per 10^5^ T cells revealed an age-related decline in line with results of previous studies. We sought to determine whether there was any difference in the sjTREC levels between individuals in whom virus was detected in the urine and those where none was found. The results, shown in Fig. 3, reveal no difference between these two groups at any age range. There have been reports of differences between thymic output and sex [34] and so we analysed sjTREC levels in males (Fig. 3b) and females (Fig. 3c) and found no difference between groups where viral DNA was detected and those where none was found.

### sjTREC and those who excrete multiple β-HHV

**Fig. 4:**
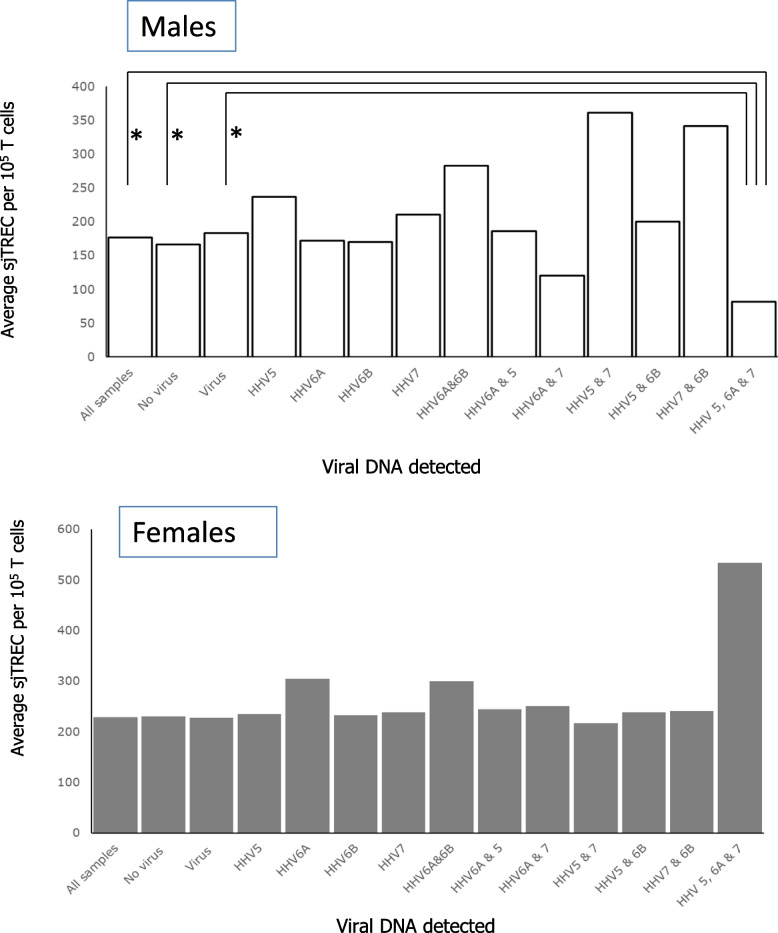
The number of sjTREC /100000 T cells in males or females comparing different combinations of virus detection where (*) indicates a significant difference between samples (*P* < 0.05)

Since the T cell arm of the immune systems should control the viral burden in an individual and there appeared to be no clear correlation between sjTREC and single viral DNA detection, we proposed that those individuals in whom multiple HHV could be detected should have some of the characteristics of measurable immune decline. To test this, we looked at sjTREC levels correlated with different viral DNA types detected and different combinations of the viral DNA detected. In males where three different viruses, HHV-5, HHV-6a and HHV-7 could be detected we saw a reduction in the average sjTREC values in individuals in this group (Fig. 4). The reduction was significant (*P* < 0.05) when this group was compared with the rest of the group (*P* = 0.013) and with the group where no virus was detected (*P* = 0.036) or the rest of the group where virus DNA was detected (*P* = 0.017). No similar correlation was found in females when we undertook this comparison (Fig. 4).

**Fig. 5:**
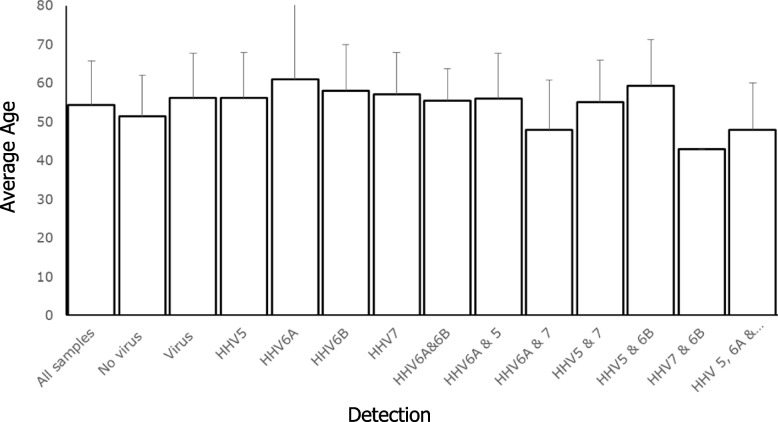
Average age of individuals in relation to the viruses detected in their urine

We were concerned that the correlation between the detection of multiple viral DNA and significantly lower sjTREC could just be a factor of the age of the individual donating the sample, as we would expect older individuals to have lower sjTREC levels and a higher likelihood of detection of virus in the urine. Our analysis of the age of those in whom multiple copies of HHV were found revealed no major difference in average age compared with the other groups (Fig. 5).

## Discussion

This is the first report in which excretion of HHV in the urine is quantified; how this varies with sex, age as well as the type of virus and linkage of this with sjTREC levels in the peripheral blood. The hypothesis driving the work was that changes in either the variety or quantity of HHV in the urine would be indicative of failures in the immune-based control of viral load. So detection and quantitation in the urine may provide a surrogate marker of immune decline and be used as a non-invasive means of immune assessment.

The β-HHV infect a large percentage of the general population often without any distinguishable clinical manifestation. They remain latent in tissues, and their reactivation is controlled by an active T-cell mediated immune response. Detection of individual HHV DNA in the urine has been used previously to identify the disease status of individuals [35] and an increase in the viral burden within the host may identify frank disease [36]. Others have suggested that the detection of virus in the urine of older individuals may be indicative of a breakdown of immune control and linked to an individual’s immune status [26].

In our study, DNA from HHV-5, 6a, 6b, and 7 could be detected in the urine of healthy individuals across the whole age range with a change in the frequency of detection only seen in males. The β-HHVs are known to be able to remain latent in the tissues and so the DNA we detect in the urine could be: fragments of viral DNA present because the cell harbouring the virus had been lysed and released its contents; complete viral particles present because they have been released from the cell because the infection has moved from a latent phase to a chronic phase; or in the urine because of being latent and inside cells of the urinary tract [37]. The detection of DNA through the release of virus from the cell from whatever cause, could result from either diminution of the T cell response as a whole or exhaustion of the viral specific T cell response. Whilst detection through the presence of the virus in cells in the urine could be due to multiple copies of virus harboured latently within the cells. From this we surmised that detection of a single virus type may not provide a robust assay of overall immunity and this was confirmed by our inability to find any significant difference in TREC levels between individuals where single virus was detected and those where no virus could be found. To some extent, this has already been validated in studies which link immune status to mortality. The OCTA and subsequent NONA studies identified HHV-5 positivity as just one of the components of the immune risk phenotype found in some older individuals with others including an altered CD4:CD8 ratio, decrease in the number of B cells, poor T cell proliferation in vitro and an increase in the number of CD8^+^CD28^− ^[38, 39].

sjTREC is both a marker of relative thymic output and also the degree of proliferation within the peripheral T cell pool [40]. We have used sjTREC as a correlate of immune competence, and not as a predictor of HHV-specific immunity. Our use of sjTREC is based in part on studies that identified a link between immune competence and sjTREC values [41] and a clear link between progressive age and declining sjTREC values [42, 43]. In addition there are longitudinal studies showing an age related decline [38] in conjunction with cross sectional studies which show the same age associated decline but also the degree of individual diversity [44] and a link with mortality [45]. A study in canines shows a clear link between sjTREC measurement and lifespan with variation due to breed type [46]. Taken together this would suggest sjTREC measurement could be a powerful tool for indicating immune competence, so the significantly lower values for sjTREC in males where three different β-HHV could be detected in the urine would strongly suggest that males possessed poorer overall T cell responses and a lower immune competence. Furthermore the identification of this trait in males, where the rate of decline in immunity is faster than in females [34, 47], would also strengthen this assertion.

Although the age-related decline in immunity is well documented, neither the rate nor the onset of the decline is uniform, but individual [48]. This provides a challenge for clinicians when determining the appropriate vaccination regimen to provide or vaccine type to administer. The availability of a simple, rapid, minimally invasive set of assays to determine immune status could assist with this.

## Data Availability

All data reported in this paper will be made available by the corresponding author on reasonable request.
